# microRNA-30b inhibits cell invasion and migration through targeting collagen triple helix repeat containing 1 in non-small cell lung cancer

**DOI:** 10.1186/s12935-015-0236-7

**Published:** 2015-09-17

**Authors:** Shanshan Chen, Ping Li, Rui Yang, Ruirui Cheng, Furui Zhang, Yuanyuan Wang, Xiaonan Chen, Qianqian Sun, Wenqiao Zang, Yuwen Du, Guoqiang Zhao, Guojun Zhang

**Affiliations:** Department of Respiratory Medicine, The First Affiliated Hospital of Zhengzhou University, Zhengzhou, 450052 China; Department of Microbiology and Immunology, College of Basic Medical Sciences, Zhengzhou University, Zhengzhou, 450001 China

**Keywords:** Non-small cell lung cancer, miR-30b, Cthrc1, Invasion, Migration

## Abstract

**Background:**

Non-small cell lung cancer (NSCLC) is the largest histological subgroup of lung cancer and has increased in prevalence in China over the past 5 years. The 5-year survival rate has remained at 15–20 %, with a median survival of 8–12 months. The tumorigenesis and progression of NSCLC is orchestrated by numerous oncogene and anti-oncogene mutations and insights into microRNA function have increased our understanding of the process. Here, we investigated the effects of miR-30b on NSCLC cell invasion and migration and explored the underlying molecular mechanisms involved.

**Methods:**

Quantitative reverse transcription PCR, wound healing assay, trans-well assays, western blotting and dual luciferase assays were performed to investigate the molecular mechanisms of miR-30b in NSCLC cells.

**Results:**

MiR-30b was down-regulated and Cthrc1 up-regulated in NSCLC tissues. Both were associated with tumor differentiation, TNM stage and lymph node metastases. Up-regulation of miR-30b restricted A549 and Calu-3 cell invasion and migration. Additionally, the expression of Cthrc1, matrix metalloproteinase-9 and matrix metalloproteinase-2 was reduced, while metallopeptidase inhibitor-1 expression increased. Bioinformatics analysis identified Cthrc1 as a target of miR-30b and western blotting and luciferase reporter assays confirmed that miR-30b regulates Cthrc1 by directly binding to its 3′UTR. Transfection of Cthrc1 without the 3′UTR restored the miR-30b inhibiting cell invasion. Up-regulation of miR-30b or down-regulation of Cthrc1 had potential significance in the invasion and metastasis of NSCLC.

**Conclusions:**

MiR-30b was down-regulated and Cthrc1 up-regulated in NSCLC tissues. Both of them were related to tumor differentiation, TNM stage and lymph node metastases. MiR-30b affected NSCLC cells invasion and migration by regulating Cthrc1.

## Background

Lung cancer is the most common cause of cancer-associated deaths worldwide [1]. Non-small cell lung cancer (NSCLC) is the largest histological subgroup, accounting for 80–85 % of all lung cancers [2]. Although substantial progress has been made in the surgical treatment, radiotherapy and chemotherapy of NSCLC [3–6], the 5-year survival rate has remained at 15–20 %, with median survival of 8–12 months [7]. The tumorigenesis and progression of NSCLC is a complex process influenced by multiple genetic factors, therefore early diagnosis and treatment is important. Preventing the tumorigenesis and progression of NSCLC at the molecular level is the key to an effective therapeutic strategy.

Insights into the oncogenic and tumor-suppressor functions of microRNAs (miRNAs) have contributed to our understanding of NSCLC tumorigenesis and progression. miRNAs are small noncoding RNA molecules that regulate the expression of at least 30 % of all protein-coding genes, influencing a variety of biological functions such as cell proliferation and survival, DNA repair and immune response [8–10]. Assorted miRNAs have been confirmed to play important roles in NSCLC. For example, miR-96 and miR-21 are significantly up-regulated in NSCLC tissues and regulate target gene expression to influence NSCLC cell migration and invasion [11, 12]. The tumor suppressor’s miR-495, miR-486-5p and miR-193a-3p/5p are all down-regulated in NSCLC tissues, contributing to the tumorigenesis and progression of the disease [13–15].

MiR-30b is a member of the miR-30 family, which plays an important regulatory role in the development of several cancers including breast cancer [16], malignant peripheral nerve sheath tumors [17], glioma [18] and lung cancer [19]. Collagen triple helix repeat containing 1 (Cthrc1) has also been associated with the development of certain cancers, including lung cancer [20–24], therefore we decided to explore a possible interaction between miR-30b and Cthrc1 and the potential significance in the invasion and metastasis of NSCLC.

In our study, we detected miR-30b and Cthrc1 expression levels in NSCLC tissues, and observed alterations in invasion, migration and expression of some metastasis associated proteins by increasing miR-30b expression in NSCLC cell lines. We next performed dual luciferase assay system and western blotting analysis on the basic of bioinformatics analysis using TargetScan and miRanda to investigated whether miR-30b regulated the expression of Cthrc1 via binding to its 3′UTR. Furthermore, co-transfection of miR-30b and pcDNA3.1-Cthrc1 can help to explore whether the effect of miR-30b can be partially restored by Cthrc1 re-expression.

## Results

### MiR-30b is down-regulated in NSCLC tissues

**Fig. 1:**
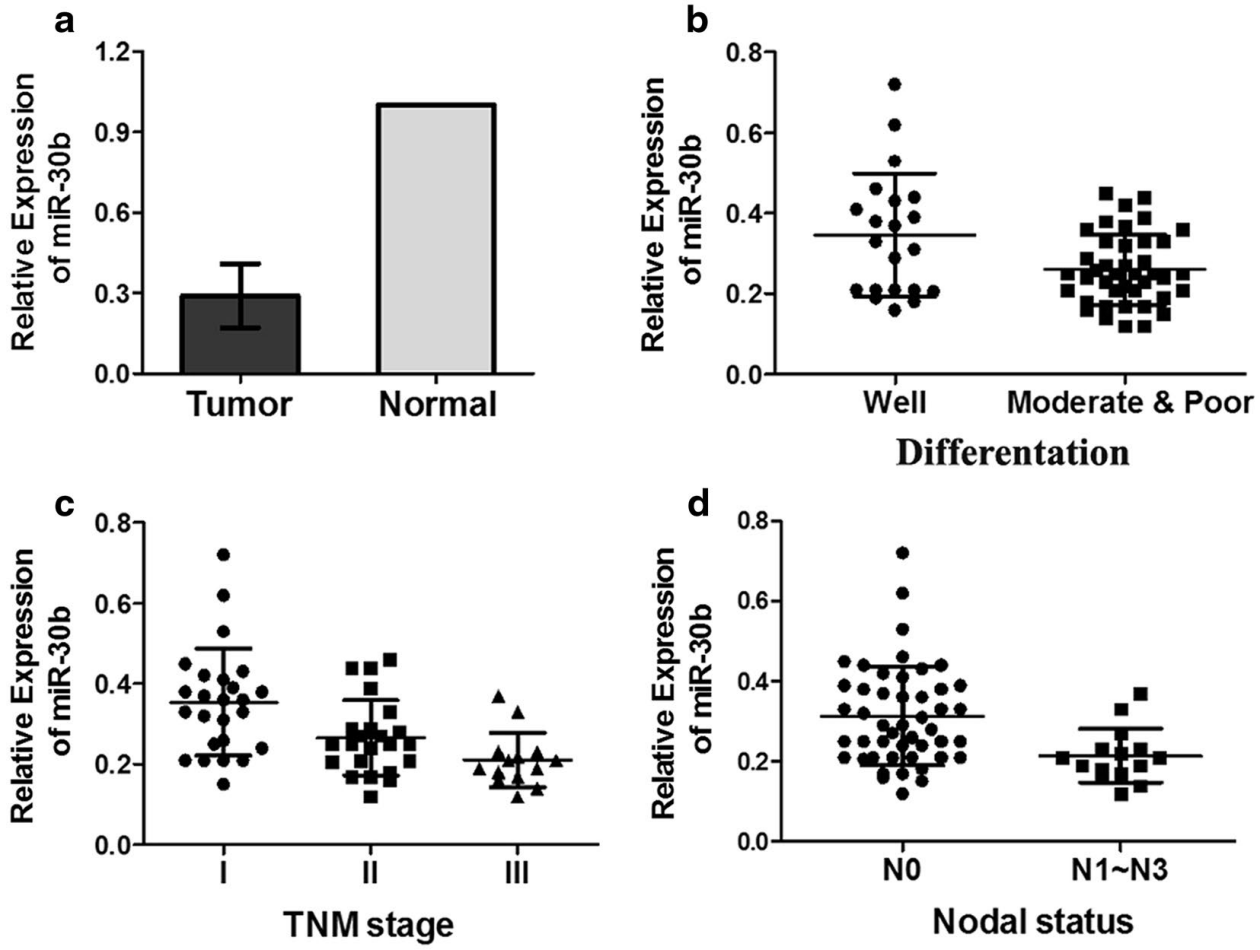
Expressions of miR-30b in paired NSCLC and adjacent normal tissues. **a** Relative expression levels of miR-30b in paired NSCLC and adjacent normal tissues. There is a statistically significant difference (*P* < 0.05). **b** On the basis of differentiation, NSCLC tissues were divided into two groups (Well and Moderate & Poor). The down-regulation of miR-30b was notably in Moderate & Poor group (*P* < 0.05). **c** In TNM stage of tumor tissues, the expression of miR-30b was successively declined (*P* < 0.05). **d** On the basis of lymph node metastases, the expression of miR-30b was significantly lower in N0 group than N1–N3 group (*P* < 0.05)

Expression of miR-30b was evaluated in 63 NSCLC and adjacent normal tissues. We found that miR-30b expression was significantly down-regulated in NSCLC tissues compared to adjacent normal tissues (*P* < 0.05, Fig. 1a). Further analysis revealed that miR-30b expression in NSCLC tissues was associated with differentiation, TNM stage and lymph node metastases (*P* < 0.05; Table 1; Fig. 1b–d). No significant differences were observed between miR-30b expression and gender, age or pathological type (*P* > 0.05; Table 1).

**Table 1:** miR-30b and Cthrc1 mRNA expression levels were associated with clinicopathological **f**eatures of NSCLC patients

Clinicopathological factor	n	miR-30b(2^−ΔΔCt)^		Cthrc1 mRNA(2^−ΔΔCt)^	
		Median ± SD	*P*	Media ± SD	*P*
Gender					
Male	38	0.2847 ± 0.1138	0.720	2.2714 ± 0.3568	0.707
Female	24	0.2960 ± 0.1307		2.2371 ± 0.3359	
Age (years)					
≥60	35	0.3081 ± 0.1324	0.156	2.222 ± 0.3971	0.345
<60	27	0.2644 ± 0.0979		2.305 ± 0.2672	
Histology					
SCC	21	0.3033 ± 0.0992	0.508	2.2548 ± 0.2960	0.957
AC	41	0.2818 ± 0.1295		2.2599 ± 0.3732	
Differentiation					
Well	21	0.3455 ± 0.1533	0.007*	2.1035 ± 0.4324	0.011*
Moderate & Poor	41	0.2602 ± 0.0870		2.3374 ± 0.2656	
TNM					
I	25	0.3540 ± 0.1327	0.000*	2.1010 ± 0.3440	0.005*
II	23	0.2663 ± 0.0927		2.3126 ± 0.2796	
III	14	0.2107 ± 0.0677		2.4494 ± 0.3470	
Lymphnode metastasis					
N0	47	0.3129 ± 0.1234	0.005*	2.1952 ± 0.3290	0.010*
N1–N3	15	0.2147 ± 0.0670		2.4555 ± 0.3352	

*SCC* squamous cell carcinoma, *AC* adenocarcinoma

* Indicated statistical significance (*P* < 0.05)

### Cthrc1 mRNA is up-regulated in NSCLC tissues

**Fig. 2:**
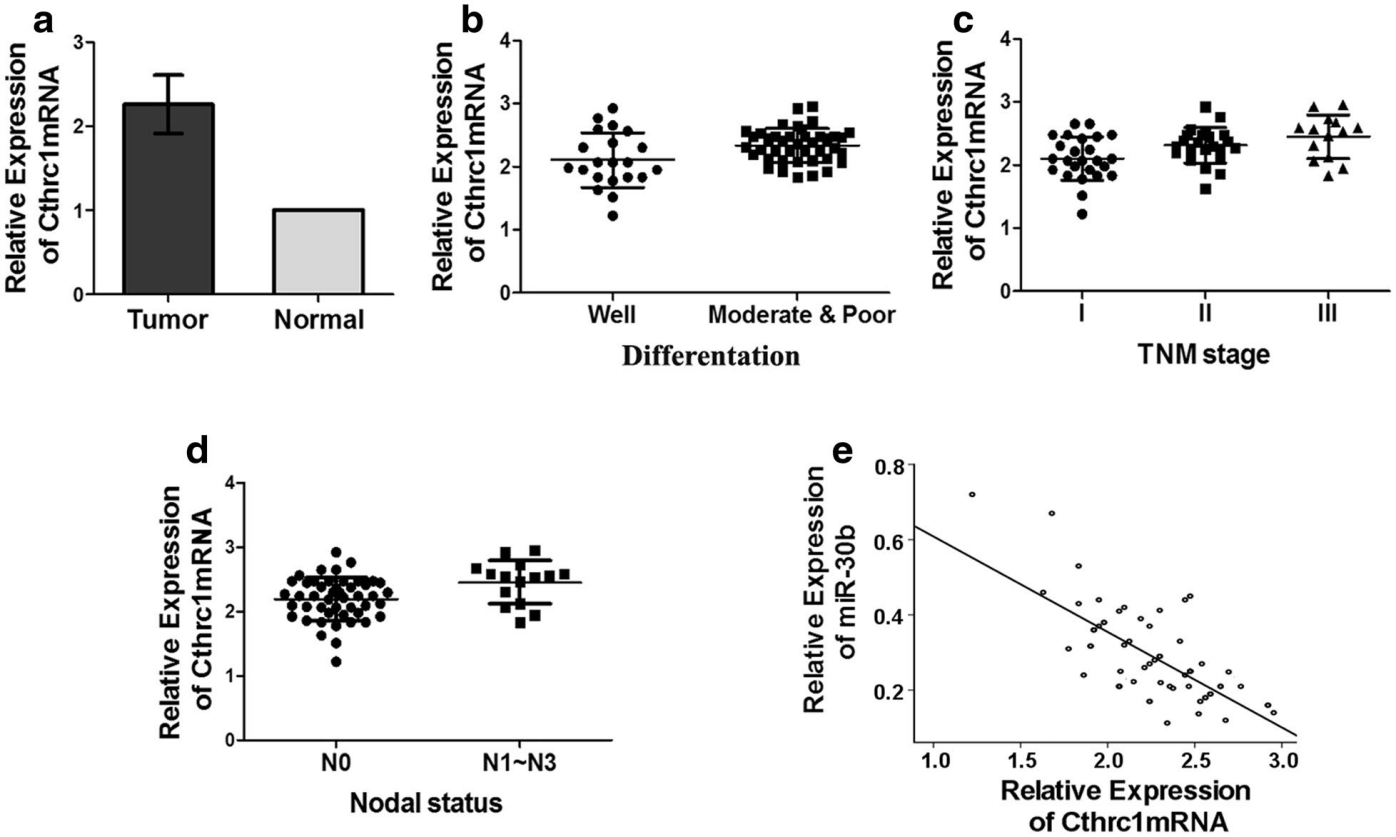
Expressions of Cthrc1 mRNA in paired NSCLC and adjacent normal tissues. **a** Relative expression levels of Cthrc1 mRNA in paired NSCLC and adjacent normal tissues. There is a statistically significant difference (*P* < 0.05). **b** On the basis of differentiation, NSCLC tissues were divided into two groups (Well and Moderate & Poor). The up-regulation of Cthrc1 was notably in Moderate & Poor group (*P* < 0.05). **c** In TNM stage of tumor tissues, the expression of Cthrc1 was successively increased (*P* < 0.05). **d** On the basis of lymph node metastases, the expression of Cthrc1 was significantly higher in N0 group than N1–N3 group (*P* < 0.05). **e** The expressions of miR-30b and Cthrc1 mRNA were negatively correlated (R^2^ = 0.734, *P* < 0.05)

Expression of Cthrc1 mRNA was also evaluated in 63 NSCLC and adjacent normal tissues. Cthrc1 mRNA expression was significantly up-regulated in NSCLC tissues (*P* < 0.05, Fig. 2a) and was also associated with differentiation, TNM stage and lymph node metastases (*P* < 0.05, Table 1; Fig. 2b–d). No significant differences were observed between Cthrc1 mRNA expression and gender, age or pathological type (*P* > 0.05; Table 1). Pearson correlation analysis indicated that the expression of miR-30b and Cthrc1 mRNA were inversely correlated (R^2^ = 0.734, Fig. 2e).

### MiR-30b inhibits the migration and invasion of NSCLC cells

**Fig. 3:**
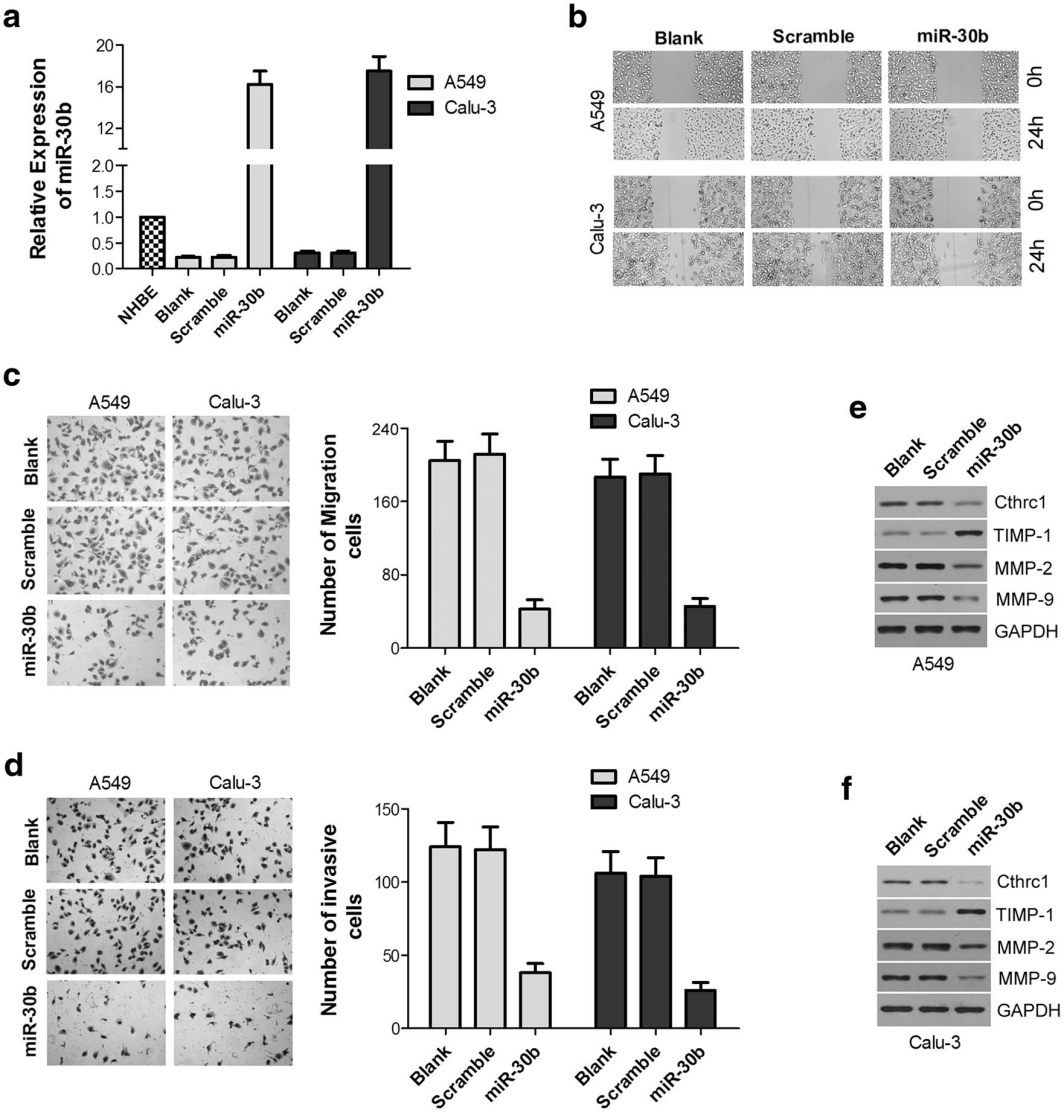
MiR-30b inhibits NSCLC cells migration and invasion. **a** After transfection of miR-30b mimics or Scramble, qRT-PCR results showed miR-30b expression was significantly increased in miR-30b group compared with NHBE group, Scramble and Blank groups (*P* < 0.05). **b** Wound healing assays were used to assess the migration ability of A549 and Calu-3 cells after transfection. miR-30b group showed a lower cell density at 24 h post-wounding compared with both Scramble and Blank groups (*P* < 0.05). **c** Transwell migration assays were used to assess the migration ability of A549 and Calu-3 cells after transfection. The numbers of A549 and Calu-3 cells in the miR-30b group passing through membrane were significantly lower compared with those from the Scramble and Blank groups (*P* < 0.05). **d** Transwell invasion assays were used to assess the invasion n ability of A549 and Calu-3 cells after transfection. The numbers of A549 and Calu-3 cells in the miR-30b group passing through the membrane and matrigel were significantly lower compared with those from the Scramble and Blank groups (*P* < 0.05).**e**, **f** Western blotting was used to analyze the expression of Cthrc1 and some other invasion-related proteins such as Cthrc1, TIMP-1, MMP-2 and MMP-9 in A549 and Calu-3 cells. The results showed that Cthrc1, MMP-2 and MMP-9 were declined but TIMP-1 was increased following transfection with miR-30b in A549 and Calu-3 cells (*P* < 0.05). *miR-30b* cells transfected with miR-30b mimics; *Scramble* cells transfected with scrambled miR-30b negative control; *Blank* cells with non-transfected

To investigate the effect of miR-30b on the migration and invasion of NSCLC cells, miR-30b mimic or a scrambled miR-30b negative control were transfected into A549 and Calu-3 cells respectively. qRT-PCR results showed miR-30b expression was significantly increased in the miR-30b group compared with the NHBE, Scramble and Blank groups (*P* < 0.05, Fig. 3a). The wound healing assay and trans-well arrays for migration ability revealed that the migration ability of A549 and Calu-3 cells in the miR-30b group was lower at 24 h post-wounding compared with both Scramble and Blank groups (*P* < 0.05, Fig. 3b, c). The cell invasion assay revealed a significant reduction in the average number of cells penetrating the trans-well membrane and matrigel in the miR-30b group (*P* < 0.05, Fig. 3d), while cells in the Scramble and Blank groups were not affected (*P* > 0.05). The expression of Cthrc1 and other invasion-related proteins such as TIMP-1, MMP-2 and MMP-9 were analyzed by western blotting. Cthrc1, MMP-2 and MMP-9 expression was reduced but TIMP-1 expression increased following transfection with miR-30b in A549 and Calu-3 cells (*P* < 0.05, Fig. 3e, f). Taken together, these findings suggest that miR-30b plays an important role in cell migration and invasion.

### MiR-30b down-regulates the expression of Cthrc1 via binding to their 3′UTR

**Fig. 4:**
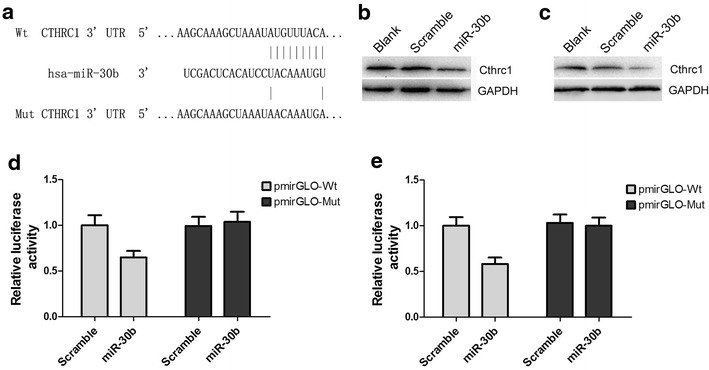
Cthrc1 was identified as a target of miR-30b in A549 and Calu-3 cells. **a** Bioinformatics analysis showed the putative binding sequence between miR-30b and Cthrc1 3′UTR. **b**, **c** Western blotting analysis of Cthrc1 expression in transfected A549 and Calu-3 cells. GAPDH was used as an endogenous control. **d**, **e** Luciferase activity determined 48 h after transfection in A549 and Calu-3 cells. In cells co-transfected with mutant-type 3′UTR of Cthrc1, no difference was found between the relative luciferase activity of cells co-transfected with miR-30b and that of the Scramble group. However, in cells co-transfected with wild-type 3′UTR, the relative luciferase activity of the cells co-transfected with miR-30b was significantly lower compared with the Scramble group. *miR-30b* cells transfected with miR-30b mimics; *Scramble* cells transfected with scrambled miR-30b negative control; *Blank* non-transfected cells

Bioinformatics analysis by TargetScan and miRanda revealed a predicted binding site for miR-30b in the 3′UTR of Cthrc1 (Fig. 4a). Furthermore, Cthrc1 expression decreased in A549 and Calu-3 cells following transfection with miR-30b mimics (Fig. 4b, c). To confirm whether Cthrc1 is a direct target of miR-30b, we performed a dual-luciferase reporter assay using luciferase-reporter vectors containing either the wild-type or the mutant 3′UTR of Cthrc1. While co-transfection with miR-30b mimics significantly decreased the luciferase activity of the reporter containing wild-type 3′UTR, it did not suppress that of the mutant-type reporter in A549 and Calu-3 cells (*P* < 0.05, Fig. 4d, e). These findings strongly suggest that miR-30b down-regulates the expression of Cthrc1 by binding to its 3′UTR.

### Expression of Cthrc1 restores miR-30b anti-invasion function

**Fig. 5:**
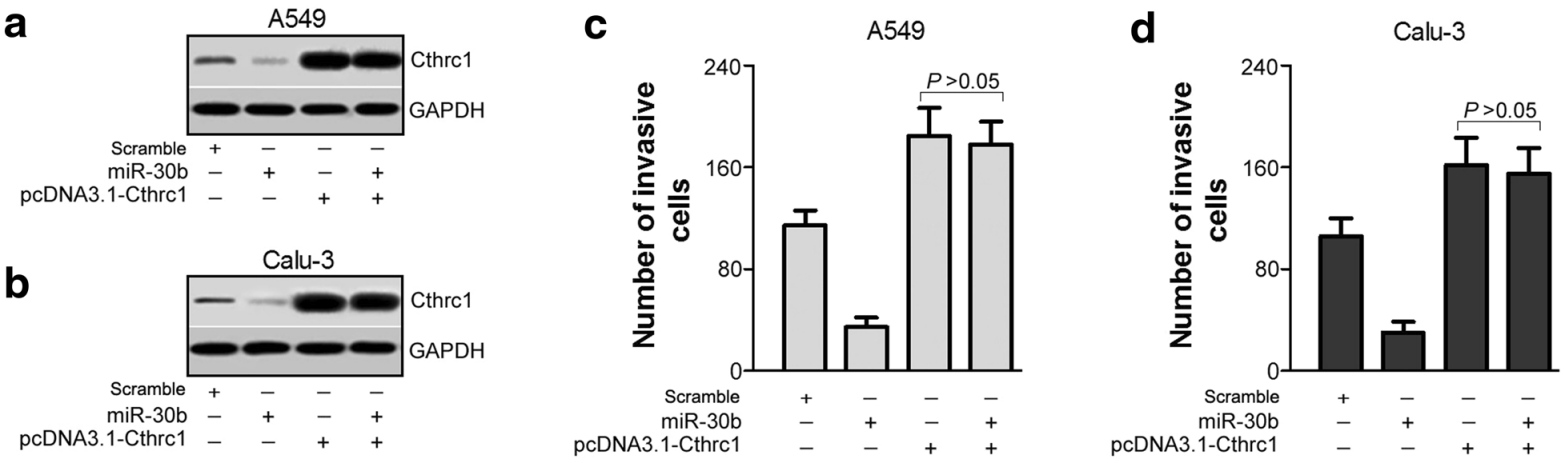
Expression of Cthrc1 restores miR-30b anti-invasion function. **a**, **b** Western blotting arrays results the Cthrc1 expression of A549 and Calu-3 cells was lower after transfection with miR-30b mimics, and was higher after transfection with miR-30b inhibitor. However, Co-transfection of miR-30b mimics and pcDNA3.1-Cthrc1 led to increased expression of Cthrc1, and abrogated the Cthrc1 expression-reducing effect of miR-30b mimics. **c**, **d** Trans-well arrays results the mean number of cells penetrating the trans-well membrane and matrigel decreased increased after transfection with miR-30b mimics, but increased after transfection with pcDNA3.1-Cthrc1 and co-transfection with pcDNA3.1-Cthrc1 and miR-30b mimics, abrogating the effect of miR-30b mimics

We next performed western blotting and trans-well assays to explore whether the effect of miR-30b can be partially restored by Cthrc1 re-expression, which also further confirm that Cthrc1 is a direct target of miR-30b. The Cthrc1 expression in A549 and Calu-3 cells were lower after transfection with miR-30b mimics, and was higher after transfection with vector containing Cthrc1 lacking the 3′UTR (pcDNA3.1-Cthrc1) into two cells. Co-transfection of miR-30b mimics and pcDNA3.1-Cthrc1 led to increased expression of Cthrc1, and abrogated the Cthrc1 expression-reducing effect of miR-30b mimics (Fig. 5a, b). For trans-well assays, the mean number of cells penetrating the trans-well membrane and matrigel decreased after transfection with miR-30b mimics, but increased after transfection with pcDNA3.1-Cthrc1 and co-transfection with pcDNA3.1-Cthrc1 and miR-30b mimics, abrogating the effect of miR-30b mimics (Fig. 5c, d). These results further suggest that Cthrc1 is a major target of miR-30b, and expression of Cthrc1 restores miR-30b anti-invasion function.

## Discussion

Lung cancer accounts for the foremost cause of malignancy-associated death worldwide, and metastasis is one of the major problems. To metastasize, a cancer cell must acquire abilities such as the capacity to colonize new tissue and evade immune surveillance, which must involve a broad spectrum of mechanisms. Gene expression profiling experiments have uncovered many genes potentially implicated in cancer invasion and metastasis [25–28]. Matrix metalloproteinases (MMPs), a family of zinc-ion dependent endopeptidases comprising more than 21 subtypes, have been widely accepted to play an important role on cancer invasion or metastasis [29–32]. In kinds of cancers, a wide spectrum of target genes are simultaneously regulated by multiple miRNAs, which leads to altered expression of invasion or metastasis-associated genes and influences the metastatic potential of many cancers [33–35]. Learning more about the cellular and molecular basis of miRNA-target gene axis in cancers will help us to understand how cancer cells disseminate and lead to new treatment strategies.

Accumulating evidences have suggested that alterations in miRNAs expression might prove crucial in cancer metastasis. In NSCLC, miR-29b was found down-regulated in high-metastatic NSCLC cells and low-expression of miR-29b in primary NSCLC tissue and correlated with lymph node metastasis [36]. In vitro and in vivo experiments, miR-425-5p contributes to invasion and metastasis of human gastric cancer [37]. In melanoma cells, miR-203 was revealed to regulate melanoma invasive and proliferative abilities in part by targeting BMI1. Our research focused on miR-30b, which has been demonstrated that enhanced the invasive capacity of melanoma cells in vitro and increased their metastatic potential in vivo [38]. MiR-30 was described as a “hub” for miRNA oncogenesis signal network in solid tumors [39]. Its up- or down- modulation has profound impacts on tumorigenesis. In our study, we found that altered miR-30b expression was associated with the occurrence of the differentiation status, TNM stage and lymph node metastases in NSCLC patients, which may be a hint for miR-30b acting as a potential tumor-suppressor contributing to the progression and metastasis of NSCLC. Nevertheless, it still needs to explore the relevant mechanism of miR-30b in NSCLC invasion and metastasis.

Naturally, many other genes as MMP family [40] and Cthrc1 [24] have been demonstrated to associate with NSCLC invasion and metastasis during the ongoing researches of NSCLC. Based on bioinformatics analysis, we detected the Cthrc1 mRNA expression and found elevated expression of Cthrc1 was also associated with the occurrence of the differentiation status, TNM stage and lymph node metastases in NSCLC patients, and Pearson correlation analysis indicated that the expression of miR-30b and Cthrc1 mRNA were inversely correlated. Furthermore, we up-regulated the expression of miR-30b in NSCLC cell lines, and found up-regulation of miR-30b restricted cell migration and invasion. Western blotting array was used to analyze the expression of invasion-related proteins and the results showed that Cthrc1, MMP-9 and MMP-2 were declined but TIMP-1 was increased following transfection with miR-30b in A549 and Calu-3 cells, which is a hint that miR-30b plays an important role in cell migration and invasion of NSCLC. Owing to the role of miRNA was realized by targeting the gene expression, the related mechanism need to be further analyzed. Bioinformatics analysis revealed that one target of miR-30b was Cthrc1. The 3′UTR region of Cthrc1 was amplified from human genomic DNA and inserted into the pmirGLO vector to construct a luciferase reporter plasmid. After transfection into A549 and Calu-3 cells, western blotting and luciferase reporter assays were performed and the results demonstrated that miR-30b regulated Cthrc1 by directly binding to its 3′UTR. Moreover, western blotting and trans-well assays further suggest that Cthrc1 is a major target of miR-30b, and expression of Cthrc1 restores miR-30b anti-invasion function.

## Conclusions

MiR-30b was down-regulated and Cthrc1 up-regulated in NSCLC tissues. Both of them were related to tumor differentiation, TNM stage and lymph node metastases. We also provided evidence demonstrating miR-30b affected NSCLC cells invasion and migration by regulating Cthrc1 expression. In the future, the miR-30b-Cthrc1 pathway could be exploited in a therapeutic approach for the treatment of NSCLC.

## Methods

### Patients and tissue specimens

Paired tumorous and adjacent normal tissues (≥3 cm away from tumor) were obtained from 63 NSCLC patients. All specimens were clinically and histologically diagnosed at the First Affiliated Hospital of Zhengzhou University between 2012 and 2014. The tumorous and adjacent normal tissues were snap-frozen in liquid nitrogen immediately after resection and stored at −80 °C until RNA extraction. This study was approved by the Ethics Committee of Zhengzhou University and written consent was obtained from all patients.

### Cell culture

Normal human bronchial epithelial cells (NHBE) and two human lung cancer cell lines (A549 and Calu-3) were used in this study (American Type Culture Collection, Manassas, VA, USA). NHBE, A549 and Calu-3 cells were cultured in Dulbecco’s modified Eagle medium (DMEM; Gibco, CA, USA) containing 10 % fetal bovine serum (FBS; Gibco), 100 U/mL penicillin and 100 μg/mL streptomycin at 37 °C with a humidified 5 % CO_2_ atmosphere.

### RNA extraction and quantitative real-time PCR

Quantitative real-time PCR (qRT-PCR) arrays were used to detect the relative levels of miR-30b and Cthrc1 mRNA in 63 paired tumorous and adjacent normal tissues. Total RNA was extracted from those frozen tissue samples containing >80 % NSCLC cells. An RNA Extraction Kit (Qiagen) was used to extract total RNA according to the manufacturer’s instructions and RNA quality was confirmed using a NanoDrop 1000 Spectrophotometer (Thermo Fisher Scientific, Wilmington, DE, USA). RNA purity was determined by an OD_260/280_ value of at least 1.8. For miR-30b expression, cDNA was obtained using the miScript Reverse Transcription Kit (Qiagen) with specific miRNA primers (Applied Biosystems). MiR-30b expression was detected using the TaqMan human microRNA assay kit (Qiagen). U6 snRNA was used as an endogenous control to normalize the data. For Cthrc1 mRNA expression, RNA was reverse-transcribed with random primers using an ABI^®^ Reverse Transcription Kit (Applied Biosystems) and qRT-PCR were performed using ABI Power SYBR^®^ Green I PCR Master Mix (Applied Biosystems) on the ABI 7500 Fast (Applied Biosystems) with glyceraldehyde-3-phosphate dehydrogenase (GAPDH) as an internal control. Data were analyzed using ABI Prism7500 SDS Software (Applied Biosystems, USA). All protocols were performed according to the manufacturers’ instructions. The comparative cycle threshold (CT) (2^−ΔΔCT^) method was used to calculate the relative expression of miR-30b and Cthrc1 mRNA.

### Western blotting

Total protein from each group was extracted and the protein concentration was measured using the Bradford DC protein assay (Bio-Rad, Hercules, CA, USA). 25 μg protein from each sample was separated using SDS–polyacrylamide gel electrophoresis (10 %) and blotted onto polyvinylidene difluoride (PVDF) filter membranes (Whatman). The membranes were blocked in 5 % skim milk for 1 h, washed four times with Tris-buffered saline containing Tween 20 (TBST) at room temperature and then incubated overnight at 4 °C with diluted primary antibodies (rabbit anti-human Cthrc1/TIMP-1/MMP-2/MMP-9 antibody, 1:500, Santa Cruz). Following extensive washing with TBST, membranes were incubated with secondary antibodies at room temperature for 1 h (goat anti-rabbit IgG, 1:1000, Santa Cruz). After four 15 min washes with TBST at room temperature, immunoreactivity was visualized by enhanced chemiluminescence. GAPDH expression (Santa Cruz Biotechnology) served as an endogenous reference.

### Cell transfection

For transfection, A549 and Calu-3 cells were seeded into 6-well plates at a density of 5 × 10^4^ cells/well. Transient transfection was performed at approximately 80 % confluence using Lipofectamine™2000 (Invitrogen, Carlsbad, CA, USA) according to the manufacturer’s instructions. 50 nM GMR-miRTM was mixed with a miR-30b mimic or scrambled miR-30b negative control (Shanghai GenePharma) in 1 µl of Lipofectamine reagent. After 24 h, qRT-PCR was used to evaluate transfection efficiencies. According to the requirements of the different experiments, A549 and Calu-3 cells were separated into three respective groups; Blank: untransfected cells, Scrambled: cells transfected with scrambled miR-30b negative control, and miR-30b: cells transfected with miR-30b mimics.

### Wound healing assay

Cell migration was analyzed using the wound healing assay. At 90 % density in 6-well plates, a sterile 200 μL pipette tip was used to scratch a straight line through the A549 and Calu 3 cell layers and the culture medium was changed. Cells were photographed 24 h after scratching. Assays were repeated three times for each clone.

### Trans-well assay

For cell invasion assay, trans-well filters (Costar) were coated with matrigel (3.9 μg/μl, 60–80 μl) on the upper surface of a polycarbonic membrane (6.5 mm diameter, 8 μm pore size). After 24 h post-transfection and then 6 h serum starvation, A549 and Calu-3 cells (2 × 10^5^) were resuspended in 200 μl of serum-free medium in the upper chambers, and while 500 μl media containing 10 % FBS (as a chemoattractant) was added to the bottom chamber. After incubating for 8 h at 37 °C in a humidified incubator with 5 % CO_2_, cells in the upper chamber were carefully removed with a cotton swab. Cells that had migrated to the basal side of the membrane were fixed with methanol, stained with hematoxylin, mounted and dried at 80 °C for 30 min. We randomly selected three visual fields and counted and recorded the number of cells invading the matrigel with an inverted microscope at 200× magnification. Each test was performed in triplicate. With the similar principle and approach, we used trans-well filters (Costar) with uncoated matrigel on the upper surface of a polycarbonic membrane (6.5 mm diameter, 8 μm pore size) for cell migration assay.

### Vector construction and the dual-luciferase reporter assay

The 3′UTR region of Cthrc1 was amplified from human genomic DNA and inserted into the pmirGLO vector (Promega, Madison, WI, USA) with XhoI and SalI at the 3′ end of the luciferase gene to construct the luciferase reporter plasmids pmirGLO-Cthrc1-Wt and pmirGLO-Cthrc1-Wt. Site-directed mutagenesis of the miR-30b target site in the Cthrc1 3′UTR was performed using a QuikChange Site-Directed Mutagenesis kit (Promega) and pmirGLO- Cthrc1-Wt as the template. For the luciferase reporter assay, A549 and Calu-3 cells were seeded into 96-well plates and transfected with 50 nM of miR-30b mimic or Scrambled and 200 ng of luciferase reporter plasmid (pmirGLO-Cthrc1-Wt/pmirGLO-Cthrc1-Mut) using Lipofectamine™2000 (Invitrogen). 48 h after transfection, cells were harvested and luciferase activity was measured with a Dual-Luciferase assay kit (Promega) according to the manufacturer’s instructions.

### Statistical analysis

All analyses were performed with SPSS 17.0 software. Normal distribution was confirmed by the normal distribution test and data were expressed as mean ± standard deviation. Significance between groups was analyzed using the student’s t test and one-way analysis of variance (ANOVA). Pearson correlation analysis was used to analyze correlation between different variable elements. *P* < 0.05 was considered statistically significant.

## Abbreviations

NSCLC: non-small cell lung cancer
miRNAs: microRNAs
Cthrc1: collagen triple helix repeat containing 1
qRT-PCR: quantitative reverse transcription polymerase chain reaction
MMPs: matrix metalloproteinases
MMP-9: matrix metalloproteinase-9
MMP-2: matrix metalloproteinase-2
TIMP-1: metallopeptidase inhibitor-1
GAPDH: glyceraldehyde-3-phosphate dehydrogenase
NHBE: normal human bronchial epithelial cell
DMEM: dulbecco’s modified eagle medium
FBS: fetal bovine serum
CT: cycle threshold
PVDF: polyvinylidene difluoride
SDS-PAGE: sodium dodecyl sulfate polyacrylamide gel electrophoresis
TBST: tris-buffered saline containing tween 20
TNM: tumor node metastasis
ANOVA: one-way analysis of variance
